# Phytochemical Characterisation of Naranjilla (*Solanum quitoense* Lam.) Segregants from Interspecific Crosses Within Section *Lasiocarpa*

**DOI:** 10.3390/molecules31132217

**Published:** 2026-06-24

**Authors:** William Viera-Arroyo, Iván Samaniego, Joseph Salazar, Michelle Noboa, Wilson Vásquez-Castillo, Jorge Merino

**Affiliations:** 1 Instituto de Investigaciones Agropecuarias (INIAP), Av. Eloy Alfaro y Av. Amazonas, Quito 170515, Ecuador; william.viera@iniap.gob.ec (W.V.-A.); ivan.samaniego@iniap.gob.ec (I.S.); michelle.noboa@iniap.gob.ec (M.N.); jorge.merino@iniap.gob.ec (J.M.); 2 Escuela Superior Politécnica de Chimborazo, Sede Orellana, El Coca 220001, Ecuador; 3 Food Chemistry Career, Faculty of Chemical Sciences, Central University of Ecuador, Quito 170521, Ecuador; jgms75@hotmail.com; 4 Grupo de Investigación en Alimentos y Agroindustria (GIAA), Ingeniería Agroindustrial, Universidad de Las Américas (UDLA), Redondel del Ciclista Vía a Nayón, Quito 170124, Ecuador

**Keywords:** carotenoids, Z-score, plant breeding, antioxidant activity, proximal analysis, *Solanum quitoense*, naranjilla, *Lasiocarpa*

## Abstract

Naranjilla (*Solanum quitoense* Lam.) is an Andean fruit crop of high economic and nutraceutical value, appreciated for its acidic-aromatic flavour and its contents of carotenoids, vitamin C and phenolic compounds. However, naranjilla production is constrained by susceptibility to pests and diseases, which has promoted the use of wild relatives from section *Lasiocarpa* in breeding programmes. The objective of this study was to characterise the physical, chemical, mineral and antioxidant attributes of 21 naranjilla materials developed in Ecuador and to identify elite segregants using multivariate approaches. Significant differences were observed among segregants for all evaluated variables. Antioxidant capacity, together with polyphenols and flavonoids, explained the largest proportion of total variation, whereas soluble solids and acidity were associated with organoleptic quality. Segregant P40 stood out due to its high antioxidant activity, soluble solids content and fruit weight; hence, it was identified as the most outstanding elite material. These results confirm the potential of naranjilla breeding in improving both yield and fruit quality.

## 1. Introduction

*Solanum quitoense* Lam., also known as naranjilla or lulo, originated in the Andes and is an economically important member of the family Solanaceae [1]. It is an Andean fruit grown in Ecuador, Colombia and mountainous regions of northern South America [2]. It has high nutraceutical value and its high antioxidant capacity provides a beneficial effect in the prevention of cancer and cardiovascular disease [3]. This species is characterised by its acidic-aromatic fruit rich in carotenoids, vitamin C and phenolic compounds, which has driven demand for the fruit in fresh and agro-industrial markets [4,5]. Naranjilla pulp is commonly consumed as juice but can also be used to create processed products [6]. Therefore, *S. quitoense* fruit has enormous potential in the agri-food industry for the development of functional foods and ingredients [3]; consequently, the fruit could serve as a base for new beverage flavours or other derivative products that could become popular in North America, Japan, Europe and similar areas [7].

However, *S. quitoense* cultivation faces challenges due to its high susceptibility to pests, such as the nematode *Meloidogyne incognita* and vascular wilt caused by *Fusarium oxysporum*, which has motivated the development of breeding strategies through crosses with species that possess genetic resistance characteristics [2,8,9]. In this context, wild relatives of section *Lasiocarpa* (including *S. hirtum*, *S. sessiliflorum*, *S. pseudolulo* and *S. quitoense* var. *septentrionale*) have been used as sources of resistance to soil pathogens [10]. These crosses generate new germplasm that produces fruit with distinct phytochemical characteristics due to the genetic variability of their parents. Consequently, the phytochemical characterisation of fruit constitutes a central component for the identification of elite materials and orientation of the selection process in genetic improvement programmes [11,12,13,14,15].

In Andean and tropical fruit trees, wide phenotypic and biochemical variability represents a strategic opportunity to generate cultivars with high nutraceutical value and commercial differentiation. In Ecuador, studies of this kind have been carried out in crops such as tree tomato (*S. betaceum*) [11], passion fruit (*Passiflora edulis*) [16,17], pitahaya (*Selenicereus megalanthus*) [18] and avocado (*Persea americana*) [19]. Nonetheless, there are other species of great potential, such as *S. quitoense*, in which genetic improvement work has been conducted through interspecific crosses with species of the *Lasiocarpa* section [20]. It is necessary to identify naranjilla fruit’s nutritional and functional compounds in order to identify promising materials, with a focus on fruit quality. Additionally, Obregón et al. [3] indicate that the nutritional profile of the fruit pulp is susceptible to changes according to the region and edaphoclimatic conditions in which they are cultivated.

The aforementioned phytochemical characterisation studies show that there is variability in the content of polyphenols, flavonoids, carotenoids, anthocyanins and vitamin C, among others, depending on the germplasm analysed [21,22]. There is great similarity in plant form and fruit appearance between *S. quitoense* L. and *S. vestissimum* D.; however, their aromas are quite different [23] and the volatile components of only the latter species have been studied extensively [24]. Currently, there are few chemical characterisation studies of naranjilla pulp. Those that do exist point out that the contents of soluble solids and acidity vary according to the ripening stage of the fruit [23]; it has also been noted that naranjilla possesses a good quantity of polyphenols [7], carotenoids (β-carotene) and volatile compounds (methyl butanoate) [25]. The high concentration of beneficial compounds in naranjilla fruit has aroused industrial interest and presents new opportunities for economic growth in the countries where it is cultivated [3]. There is evidence that the presence of certain phytochemicals in the plant confers tolerance and/or resistance to biotic factors [26].

Within this context, the objective of this study was to physically and biochemically characterise naranjilla fruit from materials derived from interspecific crosses between species of the *Lasiocarpa* section. The information generated with this type of characterisation is fundamental in order to increase the value of this crop in local and international markets as fresh fruit and as raw material for processed products.

## 2. Results

### 2.1. Physical and Chemical Traits

The analysis of variance of the fruit’s physical and chemical characteristics showed significant differences among the segregants evaluated (Table 1), allowing them to be grouped into five sets with contrasting profiles. Fruit weight showed wide variability, ranging from 41.48 g in the wild relative *Sh*.3.89 to 114.81 g in segregant T16. Firmness showed marked differences between materials, with high values in segregants such as T18 (50.98 N) and significantly lower values in segregants P37 and P18 (both with 1.78 N). Pulp yield was consistently high throughout the population, with values exceeding 92%; P30 was the segregant that reached the highest value (97.27%).

In terms of chemical quality, soluble solids ranged between 7.0 and 13.0 °Brix, with the highest values observed in the wild relative *Sh*.3.89 (13.0 °Brix) and in segregants such as P25 (12.3 °Brix). The highest titratable acidity was observed in segregant 28R3 (3.37% citric acid), while T24 had the lowest value (1.74% citric acid). The pH ranged from 3.02 to 3.54, while significant differences between groups were observed.

### 2.2. Mineral Content

The mineral content of the fruits showed statistically significant differences among segregants and groups (Table 2), evidencing marked variability in both macro- and microelements. Calcium (Ca) ranged between 0.03 and 1.17 mg 100 g pulp^−1^, with segregant T18 showing the highest content. Potassium (K) was the predominant macroelement in all segregants, ranging between 1.90 and 3.41 mg 100 g pulp^−1^, with T22 standing out (3.41 mg 100 g pulp^−1^). Iron (Fe) presented a wide range, with a lower value in P28 (19.02 mg kg^−1^) and a maximum of 45.43 mg kg^−1^ in segregant P40.

### 2.3. Antioxidant Compounds and Glycoalkaloids

The analysis of functional compounds revealed highly significant differences among the segregants evaluated (Table 3). Total polyphenol content varied widely, from values below 7 mg to a maximum of 28.03 mg GAE g^−1^, recorded in segregant P40, which stood out consistently in all antioxidant parameters. Similarly, flavonoids showed notable variation, with P40 reaching the highest value (32.41 mg catechin g^−1^). Carotenoids had a wide range, with maximum values in T18 (94.30 µg β-carotene g^−1^) and T24 (82.10 µg β-carotene g^−1^). Antioxidant capacity, measured by Ferric Reducing Antioxidant Power (FRAP) and 2,2′-azinobis (3-ethylbenzothiazoline-6-sulfonic acid) (ABTS) methods, showed patterns concordant with phenolic compound contents. Segregant P40 recorded the highest FRAP (287.83 µmol TE g^−1^ DW) and ABTS (552.77 µmol TE g^−1^ DW) values, far exceeding the rest of the materials. Vitamin C content showed considerable variability, with values ranging between 18.28 and 79.11 mg 100 g pulp^−1^ and segregant TP showing the highest concentration.

Regarding glycoalkaloids, solanine and chaconine contents varied significantly among the materials. The lowest solanine values were recorded in P29 (0.16 mg 100 g^−1^), while the highest were observed in P17 (4.35 mg 100 g^−1^). Chaconine showed a similar pattern, with particularly elevated concentrations in TP (19.60 mg 100 g^−1^).

### 2.4. Principal Component Analysis

The principal component analysis (PCA) showed a highly consistent multivariate structure among the phytochemical compounds and quality attributes evaluated. The first two components explained 79.6% of the total variance, evidencing that most of the variability among the segregants can be described in a two-dimensional space.

**Figure 1:**
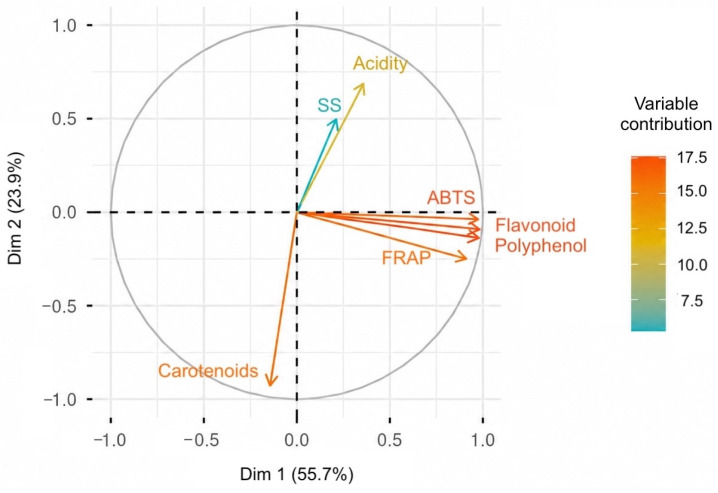
Principal component analysis (PCA) for phytochemical fruit traits in a *Solanum quitoense* breeding population. SS: soluble solids; ABTS: 2,2′-azinobis(3-ethylbenzothiazoline-6-sulfonic acid); FRAP: Ferric Reducing Antioxidant Power.

PC1, which explained 55.7% of the variation, was mainly defined by polyphenols, flavonoids, ABTS and FRAP, indicating a strong positive correlation among hydrophilic antioxidant capacity indicators. PC2, contributing 23.9% of the variability, was determined by °Brix and acidity, two variables classically associated with the organoleptic quality of the fruit. A relative correlation between carotenoids and the content of soluble solids was observed. The carotenoid content vector was oriented in an almost orthogonal direction toward the hydrophilic antioxidant group, indicating functional independence between lipophilic and hydrophilic antioxidant systems (Figure 1).

The distribution of variables in the correlation thus reveals three clearly differentiated metabolic domains: a hydrophilic antioxidant domain (polyphenols, flavonoids, ABTS and FRAP), an organoleptic domain (°Brix and acidity) and a lipophilic domain (carotenoids). This functional differentiation implies that simultaneous selection for multiple nutraceutical and sensory attributes will require multivariate strategies.

### 2.5. Z-Score and Cluster Analysis

Z-score standardisation was applied to the three variables of greatest agronomic and nutraceutical relevance: antioxidant capacity (ABTS), soluble solids (°Brix) and pulp yield. It identified the segregants whose performance was superior to the population mean (Table 4; and the full ranking results are presented in Table S1). Segregant P40 achieved the highest composite index (4.779), driven by a Z-score of 3.407 for ABTS, which markedly exceeded the ≥2 (*p* < 0.01) threshold established as the criterion for multivariate elite status, confirming its outstanding antioxidant profile. P28 (composite index: 1.544) and P25 (1.534) ranked as second-best, with positive scores across all three variables, reflecting a balanced functional and organoleptic quality profile. Segregants P39 (1.374) and T24 (1.217) completed the group of materials with positive composite indices. T24 was notable for its favourable Z-scores in soluble solids (1.258) and pulp yield (0.961), attributes of particular relevance for the fresh fruit market and agri-food processing. These findings are consistent with the multivariate structure revealed by the PCA, in which P40 was consistently set apart from the rest of the population as the genotype of greatest composite merit for the nutraceutical improvement of naranjilla.

**Figure 2:**
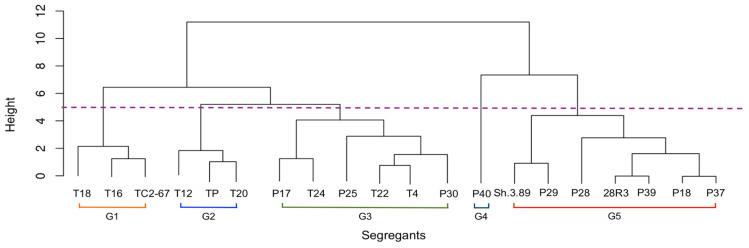
Analysis of naranjilla segregants evaluated by hierarchical cluster analysis. G: group.

The dendrogram obtained by hierarchical cluster analysis grouped naranjilla segregants according to their multivariate similarity, considering the evaluated physicochemical and phytochemical traits (Figure 2). Two main groups are identified that separate at an approximate height of 10 to 12 Euclidean distance units, indicating the presence of marked differentiation between segregant sets. The first major cluster gathers materials with relatively short distances between them, suggesting high internal homogeneity, consistent with previous observations of Colombian and Ecuadorian collections [2,5]. The second major cluster shows greater amplitude and more complex internal structure, with five subgroups formed at intermediate heights of 5 to 8 units, associated with variability inherited from crosses with *Lasiocarpa* relatives, widely used as sources of resistance or vigour [8,9]. The general structure of the dendrogram confirms that the population evaluated possesses sufficient variability for selection processes and the use of standardised indices, such as Z-scores, proved effective for identifying multivariate elites in Andean fruit species [11].

## 3. Discussion

The results reveal wide phenotypic heterogeneity among the 21 materials derived from interspecific crosses within section *Lasiocarpa*, evaluated under physicochemical, mineral and phytochemical quality criteria. This behaviour was expected because interspecific hybridisation in *Lasiocarpa* has been described as an effective pathway to expand the genetic base of naranjilla and generate novel combinations of traits, although with intense segregation in advanced generations [27]. Consistently, molecular characterisation studies in naranjilla and related species report genetic diversity and differentiation between cultivated and wild accessions, supporting the existence of elevated multivariate distances when physical and biochemical traits are integrated [9,28].

Regarding the physical attributes of the fruit, the wide variation in weight, diameters and firmness observed among segregants is in line with what has been documented in naranjilla characterisation studies under different management and postharvest conditions [29]. The high variability in pulp yield (with values consistently above 92%) is notable from an agro-industrial perspective, as this characteristic constitutes a key indicator for the commercial selection of materials, as indicated in recent reviews on the physiology of the species [30,31]. Segregant T16, which presented the highest mean weight (114.81 g), exceeds the mean values reported for the commercial variety INIAP Quitoense-2009 under field conditions in Ecuador [7]. This suggests that interspecific crosses can generate segregants with physical attributes superior to cultivated parentals [32]. Likewise, the high firmness recorded in materials such as T18 and TP could represent a competitive advantage for postharvest management and fruit shelf life [31].

The variation in soluble solids (7.0–13.0 °Brix) and titratable acidity observed among segregants is consistent with previous studies in naranjilla that document a strong influence of ripening stage and genotype on these parameters [25,33]. In particular, the wild relative *Sh*.3.89 presented the highest values of soluble solids and maturity index, which can be associated with differential sugar accumulation during fruit development, as has been described for *Lasiocarpa* section cultivars with different genetic origins [23].

The soluble solids content recorded in the evaluated segregants ranged from 7.0 °Brix (T18) to 13.0 °Brix (Sh.3.89), revealing substantial variability among groups and genetic materials. This variation is consistent with previous reports for *S. quitoense*, in which edaphoclimatic conditions, fruit maturity stage and genotype were key factors influencing the accumulation of soluble sugars [34].

Añibarro-Ortega et al. [26] reported a soluble solids content of 9.1 °Brix in *S. quitoense* fruits, a value that falls within the range observed in the present study. Several segregants, such as T12, T20, T4 and T22 (9.0–10.0 °Brix), T24 (11.5 °Brix) and P28 (12.0 °Brix), exceeded this reference value, suggesting that certain genotypes possess a greater capacity for sugar accumulation. In contrast, segregants such as T18 (7.0 °Brix), TC2-67 (7.83 °Brix) and T16 (8.5 °Brix) exhibited the lowest SS values, all below the average reported by these authors.

Segregants with the high soluble solid values, such as T24 (11.5 °Brix), combined with adequate acidity levels, present a more balanced sensory profile. This trait is of special interest for fresh fruit markets and for the development of naranjilla-based processed products [3,7]. The acidic pH range observed (3.02–3.54) is characteristic of the species and contributes to its microbiological stability, a relevant attribute for the agri-food chain [29].

In terms of functional compounds, the superiority of segregant P40 in antioxidant capacity (ABTS: 552.77 µmol TE g^−1^ DW; FRAP: 287.83 µmol TE g^−1^ DW) and in polyphenol and flavonoid contents constitutes evidence of differential accumulation of bioactive metabolites [7,35]. The naranjilla-specific literature has documented the presence of phenolic compounds (including chlorogenic acids, flavonoids and anthocyanins) and their contribution to antioxidant activity, with marked variations according to the genetic material and ripening stage evaluated [7,36].

Carotenoid content showed a metabolically independent behaviour with respect to phenolic compounds, as evidenced by the principal component analysis. Segregants T18 and T24 accumulated the highest carotenoid concentrations, a noteworthy result given that β-carotene is the main carotenoid identified in naranjilla and has been associated with antioxidant properties and as a vitamin A precursor in human diets [7,9]. The variation in carotenoids among segregants can be explained by differences in gene expression related to the biosynthesis of these lipophilic compounds, which in *Solanum* is strongly genotype-dependent and can be modulated by edaphoclimatic conditions [3]. The functional independence observed between the hydrophilic antioxidant domain (polyphenols, flavonoids, ABTS and FRAP) and the lipophilic domain (carotenoids) implies that simultaneous selection for multiple nutraceutical attributes will require multivariate strategies, as employed in this study [11].

The vitamin C content showed considerable variability among the segregants evaluated, with TP standing out as the material with the highest concentration. The values recorded are comparable with those reported for naranjilla in Ecuador by Vasco et al. [36], who identified this species as one of the richest sources of vitamin C among the fruits that they studied. Variability in vitamin C among the evaluated materials could be associated with both genetic differences and the ripeness of the fruit at harvest, factors that modulate ascorbic acid biosynthesis [3,23]. This attribute has high nutraceutical value since 100 g of fresh naranjilla fruit can provide a significant fraction of the daily recommended amount of vitamin C [36].

Antioxidant capacity values measured by ABTS and FRAP across the evaluated segregants exhibited substantial inter- and intragroup variability. ABTS values ranged from 95.74 µmol TE g^−1^ DW (T24) to 552.77 µmol TE g^−1^ DW (P40), while FRAP values ranged from 86.75 µmol TE g^−1^ DW (P29) to 287.83 µmol TE g^−1^ DW (P40), positioning the latter as the segregant with the highest antioxidant potential across both assays. Most segregants exhibited intermediate ABTS values of 130 to 270 µmol TE g^−1^ DW. In Colombia, Contreras-Calderón et al. [37] reported FRAP and ABTS values of 69.79 ± 0.52 and 125.77 ± 8.76 µmol TE g^−1^ DW, respectively, which are notably lower than those recorded for most segregants in the present study, suggesting that the evaluated germplasm harbours genotypes with comparatively high antioxidant potential. In addition, Andrade-Cuvi et al. [38] reported an ABTS value of 445.5 µmol TE g^−1^ DW, a threshold exceeded in our study only by segregant P40 (552.77 ± 6.00 µmol TE g^−1^ DW), while the remaining segregants performed below this reference. In contrast, Moreno et al. [39] reported a substantially higher FRAP value of 618.56 µmol TE g^−1^ DW for *S. quitoense* pulp, considerably exceeding even P40 (287.83 µmol TE g^−1^ DW) in our study and underscoring the wide intraspecific variability documented for this trait. For Peruvian naranjilla, Obregón et al. [40] reported ABTS and FRAP values of 91.55 and 20.31 µmol TE g^−1^ DW, respectively, which are below the range observed across most segregants in our study. These discrepancies likely reflect the combined influence of genetic background, geographic origin, edaphoclimatic conditions, maturity stage at harvest and methodological differences among studies, all recognised as key determinants of antioxidant activity variation in species of the *Lasiocarpa* section. Nevertheless, the consistent identification of P40 as the top-performing segregant across both ABTS and FRAP variables reinforces its relevance as a priority genotype for breeding programmes aimed at improving the functional quality and antioxidant properties of naranjilla. These results reveal a wide range of antioxidant potential within the evaluated germplasm and suggest that genetic factors play an important role in determining antioxidant capacity in naranjilla pulp.

Recent studies confirm that the pulp, peel and seeds of *S. quitoense* contain significant quantities of phytochemicals with antioxidant and antimicrobial activity, with inter-tissue differences emphasising the importance of including different fruit fractions in comprehensive nutraceutical evaluations [41]. The high concentration of bioactive phenolic compounds in naranjilla has generated increasing industrial interest concerning the development of functional foods and pharmaceutical products [3]. This reinforces the strategic value of identifying segregants with differentiated antioxidant profiles such as P40.

Regarding glycoalkaloids, solanine and chaconine showed variable concentrations among the evaluated materials. These compounds are steroidal secondary metabolites characteristic of the genus *Solanum*, with a dual functional role: at the plant level, they confer resistance to pathogens and soil pests [42,43], while at the dietary level they present anticancer, anticholesterolemic, antimicrobial and anti-inflammatory properties at controlled doses, although they can also induce toxicity at elevated concentrations [44]. There are no reported maximum levels of glycoalkaloids for naranjilla; however, in potato (a species belonging to the same Solanaceae family), a maximum limit of 200 mg kg^−1^ has been reported. The results obtained in our study for naranjilla are below the limit mentioned for potato and thus it may not represent a risk to human health.

In the context of naranjilla improvement through crosses with wild relatives of section *Lasiocarpa*, it is particularly relevant to monitor glycoalkaloid content in progeny since cultivars derived from wild species can have higher levels of these compounds compared to cultivated varieties [44,45]. Segregants P39 and P29, which showed the lowest solanine values, are of special interest from a food safety perspective. This information is fundamental in order to guide selection in breeding programmes aimed at ensuring both the nutraceutical quality and food safety of the fruit [9].

The mineral content of the naranjilla fruits varied significantly in both macroelements and microelements. K was the predominant macroelement in all segregants, in accordance with the mineral profile described for other Andean tropical fruits [26]. Segregant P40, in addition to excelling in antioxidant compounds, exhibited the highest Fe content (45.43 mg/kg), reinforcing its positioning as an elite material from an integral nutraceutical perspective. The variability in zinc (Zn), Ca and microelement contents among segregants may be linked to differences in mineral absorption efficiency derived from the genetic diversity of the parentals used in the crosses [28] as well as interactions with the edaphic substrate, as has been documented in other Andean solanaceous species [16].

Regarding K, Añibarro-Ortega et al. [26] reported a value of 4732 mg 100 g^−1^ DW, which exceeds the range observed among our evaluated segregants (up to 3410 mg 100 g^−1^ DW), with T22 exhibiting the highest concentration. These differences may reflect both the genotypic origin of the material and the edaphoclimatic conditions under which it was produced. For Ca, the value found by Añibarro-Ortega et al. [26] (49.5 mg 100 g^−1^ DW) was greatly surpassed by T18 (1170 mg 100 g^−1^ DW) and T20 (580 mg 100 g^−1^ DW) in our study, whereas most of the remaining segregants displayed lower concentrations (30–110 mg 100 g^−1^ DW), highlighting substantial intraspecific variability in the accumulation of this macronutrient. The magnesium (Mg) value recorded by Añibarro-Ortega et al. [26] (193.8 mg 100 g^−1^ DW) exceeded the concentrations recorded in all segregants (90–180 mg 100 g^−1^ DW) in our study, which may be associated with differences in soil Mg availability and genotypic variation in root uptake efficiency. Turning to sodium (Na), the value observed by Añibarro-Ortega et al. [26] (41.2 mg 100 g^−1^ DW) was higher than that of most evaluated segregants in our study, which ranged from 0 to 40 mg 100 g^−1^ DW, with only T12 (40 mg 100 g^−1^ DW) approaching this level.

The same authors stated that in terms of Fe, the value (2.68 mg 100 g^−1^ DW) was within the range observed among our materials, with P40 exhibiting the highest concentration (4.54 mg 100 g^−1^ DW), making it a genotype of particular nutritional interest. The Zn value in Añibarro-Ortega et al. [26] (1.86 mg 100 g^−1^ DW) was exceeded by T16 (2.35 mg 100 g^−1^ DW) and T12 (2.11 mg 100 g^−1^ DW) in our study, suggesting a differential capacity for Zn accumulation in these genotypes. The manganese (Mn) concentration in Añibarro-Ortega et al. [26] (0.81 mg 100 g^−1^ DW) was generally higher than the values observed in our segregants, although TC2-67 (0.41 mg 100 g^−1^ DW), T16 (0.49 mg 100 g^−1^ DW) and P25 (0.49 mg 100 g^−1^ DW) exhibited comparatively elevated levels within the collection. Finally, the copper (Cu) value in Añibarro-Ortega et al. [26] (1.28 mg 100 g^−1^ DW) was not exceeded by any segregant in our study; however, T20 (1.20 mg 100 g^−1^ DW) showed a comparable concentration, while TC2-67 (0.749 mg 100 g^−1^ DW) and T16 (0.800 mg 100 g^−1^ DW) also presented relatively high levels.

Overall, these results indicate that although previous studies have reported higher concentrations of certain macroelements, particularly K and Mg, several segregants (T18, T16, T12 and P40) displayed outstanding mineral profiles for specific elements. This finding highlights the potential of the diversity present within species of the *Lasiocarpa* section for breeding programmes focused on nutritional value.

The PCA revealed, as aforesaid, that 79.6% of the total variance could be explained by the first two components, which is considered high [46]. PC1, mainly defined by hydrophilic antioxidant capacity indicators (polyphenols, flavonoids, ABTS and FRAP), explained 55.7% and PC2, defined by organoleptic quality variables (°Brix and acidity), explained 23.9%. This variation structure is consistent with what has been observed in other Andean solanaceous species, where phenolic compounds and antioxidant capacity are the main descriptors of phytochemical diversity among materials of the same genetic collection [11]. The separation of three independent metabolic domains (hydrophilic antioxidant, organoleptic and lipophilic) underscores the complexity of simultaneous selection for multiple quality attributes and confirms the need for multivariate tools [47] to identify elite materials in naranjilla-segregating populations, as aforementioned.

The use of Z-scores allowed the standardisation of each trait with respect to the population mean, facilitating comparisons between variables with different units and enabling the simultaneous identification of outstanding genotypes in multiple attributes [48,49]. This methodology has been validated in various Andean and tropical fruit species for selecting promising materials in terms of yield, quality and bioactive metabolite content, as in passion fruit (*P. edulis*) [17] and tree tomato (*S. betaceum*) [11]. In the present study, segregant P40 was the best-performing elite material because it clearly showed significantly higher values for polyphenols, flavonoids and antioxidant capacity by ABTS and FRAP. This may indicate that polyphenols and flavonoids are mainly responsible for the antioxidant capacity. This segregant also showed high contents of Fe, K and soluble solids. Its clear multivariate differentiation in the dendrogram and Z-scores ≥ 2 in key variables consolidate it as the most promising candidate for advanced selection cycles or for use as a progenitor in crosses aimed at improving the nutraceutical quality of the fruit. Segregants P28 and P25, with balanced physicochemical quality and antioxidant stability profiles, emerged as moderately elite candidates, relevant for both commercial selection and recurrent improvement programmes aimed at increasing the functional value of naranjilla in local and international markets [3,9].

## 4. Materials and Methods

### 4.1. Plant Material

Fresh fruit from 21 materials derived from crosses between parental *Solanum quitoense* plants and other wild relatives of section *Lasiocarpa* (Table 5), included in the Instituto Nacional de Investigaciones Agropecuarias (INIAP) breeding programme, were evaluated in this study. Fruits were harvested after 270 days of transplanting. They were collected when the colour change (green to orange) was 75%, according to harvest criteria described by Andrade-Cuvi [35]. Twenty fruits per segregant were taken from the field and carried to the laboratory; the pulp was obtained and immediately lyophilised for the analysis.

### 4.2. Physicochemical Determinations

The physicochemical parameters established for naranjilla quality studies were evaluated, namely weight, polar and equatorial diameters, firmness, pulp yield, soluble solids (°Brix), pH, titratable acidity and maturity index.

#### 4.2.1. Weight

Fruit weight was measured using a digital balance (BBC31, Boeco, Hamburg, Germany) and recorded in grams (g).

#### 4.2.2. Firmness

The penetration force required to penetrate the fruit pulp was measured directly using a digital penetrometer (FR-5120, Lutron, Coopersburg, PA, USA) and the result was expressed in Newtons (N).

#### 4.2.3. Yield

Plant yield was calculated from the number of fruits per plant and their fresh weight. Fruit weight was measured using a digital balance (BBC31, Boeco, Hamburg, Germany) and recorded in grams (g). Peel, pulp and seed yields were determined by separately weighing each fruit fraction and calculating their relative contribution using the following equation:

$$X\;=\;\frac{Wp}{Wf}\times \;100$$

where *X* is the percentage of each fruit part (peel, pulp or seed), *Wp* is the weight of each fruit part (peel, pulp or seed) and *Wf* is the total fruit weight.

#### 4.2.4. Soluble Solids (°Brix)

The total soluble solids concentration was determined by refractometry using an ATAGO digital refractometer (Tokyo, Japan), according to the methodology specified by AOAC 2000 [50]. Two drops of fruit juice were placed on the prism of the equipment surface and the percentage of soluble solids was shown directly on the equipment screen, expressed in terms of °Brix.

#### 4.2.5. Titratable Acidity

The titratable acidity content of the fruits was measured by potentiometric titration with the help of a standardised alkaline solution [50]. A total of 30 g of fruit pulp was weighed and washed with distilled water at a volume of 200 mL. Subsequently, 20 mL was placed in a 25 mL beaker and titrated with a 0.1 N NaOH solution until pH 8.2 was reached. The results are reported based on the predominant organic acid in the sample, which in this case was citric acid.

#### 4.2.6. Maturity Index

The maturity index of the segregants was determined using the relationship between the TSS content and the titratable acidity (*TA*) [50] according to the following equation:

$$MI\;=\;\frac{TSS}{TA}$$

where *MI*: maturity index, *TSS*: total soluble solids (°Brix) and *TA*: titratable acidity (g citric acid/100 g).

### 4.3. Preparation of Samples for Analysis

Fruit pulp was obtained then packed and sealed into plastic bags and stored in a freezer (−12 °C) for subsequent drying by lyophilisation. The dried samples were subject to a grinding process in a Retsch model ZM 200 mill (Hann, Germany) then passed through a stainless-steel sieve (1 mm mesh) to ensure a uniform particle size. Lastly, the samples were packed in plastic containers with hermetic lids then labelled and protected from light until analysis.

### 4.4. Mineral Analytical Procedures

For mineral determination, 2 g of lyophilised sample were weighed into a 25 mL porcelain crucible and calcined at 600 °C for 12 h. After this time, the samples were cooled in a desiccator. Subsequently, 10 mL of Type I water and 5 mL of concentrated hydrochloric acid were added to each crucible containing the ash and the mixture was digested on a hot plate at 100 °C until the acid volume was reduced by half. The digested samples were filtered into 100 mL volumetric flasks and diluted to volume with Type I water.

Phosphorus (P) content was determined by mixing 0.5 mL of sample with 4.0 mL of Type I water and 0.5 mL of a 1% ammonium molybdovanadate solution. After thorough homogenisation, absorbance was recorded using a UV–visible spectrophotometer (UV2600, Shimadzu, Tokyo, Japan). P concentration was quantified from a calibration curve and expressed as mg 100 g^−1^ DW.

For macronutrient analysis, 4.5 mL aliquots of each sample were transferred into test tubes. To determine Ca and Mg, 0.5 mL of a 1% lanthanum solution was added to minimise analytical interferences. In terms of Na and K determinations, the same sample volume was supplemented with 0.5 mL of a 1% lithium solution as an interference suppressor. The micronutrient analysis for Zn, Cu, Fe and Mn was performed using 5.0 mL of sample without the addition of interference-correcting reagents [11]. Absorbance measurements of all prepared solutions were obtained using an atomic absorption spectrophotometer (AA7000, Shimadzu, Tokyo, Japan). The concentrations of macronutrients (Ca, Mg, Na and K) and micronutrients (Cu, Fe, Mn and Zn) were quantified using calibration curves for each element and were expressed in mg 100 g pulp^−1^ and mg kg pulp^−1^, respectively.

### 4.5. Functional Compounds

The functional characterisation of the fruit was performed by quantifying total polyphenols, flavonoids, carotenoids and antioxidant capacity using FRAP and ABTS methods. These determinations were performed using UV–visible spectrophotometry. Calibration curves were prepared in triplicate over three consecutive days for antioxidant capacity and functional compounds, while high-performance liquid chromatography (HPLC) analyses were validated using triplicate curves in one day.

#### 4.5.1. Extraction of Functional Compounds

To extract antioxidant compounds, polyphenols and flavonoids, 0.3 g of dry sample was placed in 15 mL polyethylene centrifuge tubes. To the tubes, 5 mL of the extraction solution (methanol/water/formic acid, 70/30/0.1 *v*/*v*/*v*/*v*.) was added and homogenised by shaking for 5 min in a Mistral 4600 vortex apparatus (Vernon Hills, IL, USA). The samples were then placed in a Cole-Parmer 8892-MTH ultrasonic bath (Vernon Hills, IL, USA) and centrifuged at 5000 rpm for 10 min. The supernatant was placed in a 25 mL amber balloon and this extraction procedure was repeated four times continuously. This extraction process was used to quantify the antioxidant capacity of the samples.

#### 4.5.2. Polyphenols

Total polyphenol content quantification was carried out by UV–visible spectrophotometry [13]. The diluted extract (1 mL) was placed in a 15 mL test tube and 6 mL of distilled water and 1 mL of Folin–Ciocalteu reagent were added, then the mixture was left to rest for 3 min. After that, 2 mL of 20% Na_2_CO_3_ (*w*/*v*) was added and heated to 40 °C for 2 min. The absorbance of the blue chromophore was measured at 760 nm using a UV–visible spectrophotometer (Model 2600, Shimadzu). Five extraction cycles were necessary in order to enable total polyphenol content recovery. Quantification was carried out by interpolating the corresponding absorbance value of each sample on a calibration curve made with gallic acid at 0–100 mg gallic acid/L. Results were expressed as mg of gallic acid equivalents per gram of dry sample (mg GAE g^−1^ DW).

#### 4.5.3. Flavonoids

Total flavonoid content was determined according to the method described by Zhishen et al. [45] using a UV–visible spectrophotometer (Model 2600, Shimadzu). Briefly, 1 mL of diluted extract was transferred into a 15 mL test tube containing 4 mL of distilled water and thoroughly mixed. Subsequently, 0.3 mL of 5% (*w*/*v*) sodium nitrite was added, followed by a 5 min incubation period. Then, 0.3 mL of 10% (*w*/*v*) aluminium chloride was incorporated and the mixture was allowed to stand for an additional 5 min. Afterwards, 2 mL of 1 N Na(OH) was added and the final volume was adjusted to 10 mL with distilled water. The absorbance of the resulting pink-coloured chromophore was measured at 490 nm using a UV–visible spectrophotometer (Model 2600, Shimadzu).

A total of five extraction cycles were performed to ensure complete recovery (100%) of flavonoids. Quantification was achieved by interpolating the absorbance values of the samples against a calibration curve prepared with (+)- catechin standards ranging from 0 to 100 mg (+)- catechin L^−1^. The results were expressed as milligrams of catechin equivalents per gram of dry weight (mg catechin g^−1^ DW).

#### 4.5.4. Carotenoids

Total carotenoid content was determined according to the methodology reported by Llerena et al. [34]. All procedures were carried out under conditions that minimised exposure to light and oxygen. For the analysis, 1.0 g of freeze-dried sample was extracted with 50 mL of a solvent system consisting of hexane, ethanol and acetone in a 50:25:25 (*v*/*v*/*v*) ratio, supplemented with 0.1% (*w*/*v*) butylated hydroxytoluene (BHT) and 5 g calcium chloride (*w*/*v*). Each component was added sequentially to the sample.

The extraction mixture was homogenised for 20 min in a refrigerated water bath maintained at 4 °C. Phase separation was then promoted by gradually adding 15 mL of distilled water over a period of 10 min. The resulting extract was filtered and transferred to a separatory funnel, from which the organic phase was collected into a volumetric flask. Hexane was subsequently added to adjust the final volume to 50 mL. Total carotenoid concentration was quantified by measuring absorbance at 450 nm using a UV–visible spectrophotometer (Model 2200, Shimadzu). Results were expressed as micrograms of β-carotene per gram of pulp dry weight (µg β-carotene g^−1^ DW). The *TCC* was calculated using the following equation:

$$TCC\;=\frac{A\;\times \;VT\;\times \;10^{4}}{2592\;\times \;W}$$

where *A* represents the absorbance measured at 450 nm, *VT* is the total extract volume, 10^4^ is the conversion factor expressed in µg g^−1^, 2592 corresponds to the molar extinction coefficient of β-carotene in hexane and *W* is the sample weight.

#### 4.5.5. Antioxidant Capacity Using the ABTS Method

Antioxidant activity was determined using the ABTS•+ (2,2′-azinobis(3-ethylbenzothiazoline-6-sulfonic acid)) radical cation decolourisation assay, following the methodology described by Samaniego et al. [13]. The ABTS•+ radical solution was generated by mixing ABTS (7 mM) and potassium persulfate (2.45 mM) solutions at a 1:1 (*v*/*v*) ratio. After standing for 24 h, the absorbance of the resulting ABTS•+ solution was measured and adjusted with phosphate buffer to obtain an absorbance of 1.1 ± 0.01 at 734 nm.

For the assay, 200 µL of sample was transferred into 15 mL test tubes and mixed with 3.8 mL of the ABTS•+ working solution. The reaction mixture was allowed to stand for 45 min before absorbance was recorded at 734 nm using a UV–visible spectrophotometer (Model 2600, Shimadzu). Antioxidant activity was quantified by interpolating the absorbance values of the samples against a calibration curve previously prepared with Trolox standards ranging from 0 to 800 µmol Trolox L^−1^. Results were expressed as micromoles of Trolox equivalents per gram of dry sample weight (µmol TE g^−1^ DW).

#### 4.5.6. Antioxidant Capacity Using the FRAP Method

FRAP was evaluated to determine antioxidant activity according to the method described in [13]. Briefly, 1 mL of diluted extract was transferred into a 15 mL test tube, followed by the addition of 2.5 mL of phosphate buffer (pH 6.6) and 2.5 mL of 1.0% potassium ferricyanide solution. The mixture was thoroughly mixed and incubated at 50 °C for 20 min. Subsequently, 2.5 mL of 10% trichloroacetic acid, 2.5 mL of distilled water and 0.5 mL of 1% FeCl_3_ solution were added. The reaction mixture was homogenised using a vortex mixer (Mistral Multi-Mixer, Melrose Park, IL, USA) and then allowed to stand in the dark for 30 min to facilitate the formation of the green-coloured ferrous chloride–potassium ferrocyanide complex. Absorbance was measured at 700 nm using a UV–visible spectrophotometer (Model 2600, Shimadzu). Antioxidant activity was quantified following the same procedure used for the ABTS assay and the results were expressed as µmol TE g^−1^ DW.

#### 4.5.7. Vitamin C

Vitamin C (ascorbic acid) was measured using a reflectometer (RQflex plus 10, Merck, Darmstadt, Germany). Firstly, 30 g of pulp was weighed and made up to 200 mL with distilled water. A 20 mL aliquot was taken and tested by immersing an ascorbic acid strip in the reflectometer. The final result was expressed in mg 100 g pulp^−1^ FW, according to the following formula:

$$Vitamin\;C=\frac{L\;\times \;V}{Sw}$$

where *L* = reflectometer lecture (mg/L^−1^), *V* = final volume (mL) and *Sw* = sample weight (g).

### 4.6. Glycoalkaloid Analytical Procedures

Glycoalkaloids (α-solanine and α-chaconine) were determined via HPLC using the methodology proposed by Attoumbré et al. [51] with certain modifications. Briefly, 5 g of lyophilised and pulverised sample was weighed in a 250 mL Erlenmeyer flask, 200 mL of extracting solution methanol/water/acetic acid (73/18/9 *v*/*v*/*v*) was added and this was stirred at 400 rpm for 15 min and subsequently at 900 rpm for 1 h. The extract was filtered through Whatman No. 41 paper and concentrated by rotary evaporation to approximately 20 mL. The extract was transferred quantitatively to 250 mL ultracentrifuge tubes, washed with 75 mL of methanol and 75 mL of 25% NH_4_OH was added; the mixture was agitated vigorously and allowed to stand for 45 min at 5 °C, then centrifuged at 6000 rpm and the supernatant was discarded. The precipitate was dissolved with 5 mL HPLC-grade methanol, filtered through 0.22 μm Millipore membranes and placed in 2 mL amber vials for injection. The chromatographic conditions were: Hypersil GOLD C18 column (Thermo Fisher Scientific, Waltham, MA, USA) (5 μm × 4.6 mm × 250 mm); mobile phase: acetonitrile/bidistilled water/phosphate buffer pH 7.6 (60/30/10 *v*/*v*/*v*); flow: 1.25 mL/min; UV–visible detector at 202 nm. Quantification was performed by comparing the samples’ peaks with their respective standards and the results were expressed as mg 100 g^−1^.

### 4.7. Statistical Analysis

For the statistical analysis, each segregant (including the wild relative) was considered as a treatment. A completely randomised design was applied and all measurements and analyses were made in triplicate. Ten fruits per replication were used for each treatment to measure physical variables such as fruit weight and firmness. Turning to the chemical variables, three replications per treatment were carried out for soluble solids and titratable acidity. For the analysis of minerals, functional compounds and glycoalkaloids, the experimental unit was a 20 g sample of freeze-dried pulp.

An analysis of variance was performed using the segregant as the independent factor, preceded by normality and homoscedasticity tests (Levene and Bartlett). Variables showing significant differences were subjected to Tukey’s test (5%) to determine differences between means and ranges. A multivariate principal component analysis was also performed to determine the variables that explain the variability of the data and their relationship. Subsequently, Z-scores were estimated to select individuals showing a value of ≥2 (*p* < 0.01) for the independent desirable characteristics using pulp yield, ABTS content and soluble solids content. A cluster analysis was performed to classify the segregants into groups based on their similarity in terms of the characteristics evaluated. These analyses were carried out using R package version 4.6.0.

### 4.8. Limitations of This Study

This study was designed as an assessment to generate baseline information for the selection of promising naranjilla segregants with desirable traits relating to fruit quality (physiochemical and mineral composition). Consequently, a detailed characterisation (profile) of antioxidant compounds, such as polyphenols, flavonoids and carotenoids, was not conducted at this stage and this must also be performed in the elite individuals identified in this study.

## 5. Conclusions

Interspecific hybridisation within section *Lasiocarpa* of *Solanum* effectively expands the phenotypic and biochemical diversity of naranjilla beyond the range observed in commercial varieties of *S. quitoense*, generating a segregating population with sufficient genetic variance to support simultaneous selection for fruit quality, nutraceutical value and mineral composition. This finding validates the dual-objective breeding strategy, in which the transfer of pathogen resistance from wild relatives is pursued without compromising the physicochemical and functional attributes that determine the commercial acceptance and agro-industrial potential of the species.

The integration of multivariate statistical tools, such as the principal component analysis, hierarchical cluster analysis and Z-score-based selection indices, proved to be a robust and transferable framework for identifying elite genotypes in a population where quality attributes are governed by functionally independent metabolic domains. This methodological approach, previously validated in other Andean fruit species, is hereby confirmed as applicable to naranjilla-segregating populations derived from broad crosses.

Among the evaluated segregants, P40 was consolidated as the elite material with the greatest composite merit. Its values were consistently greater than the population means in terms of antioxidant capacity, phenolic compounds and key mineral content, making it the priority candidate for advanced agronomic trials and eventual release as an improved variety with superior nutraceutical properties. Segregants P28 and P25, with balanced physicochemical and functional quality profiles, are suitable as progenitors in recurrent improvement cycles, while materials with low glycoalkaloid content are of special interest for the fresh fruit market since this low content is increasingly relevant for compliance with food safety standards in export markets.

## Figures and Tables

**Table 1:** Physical and quality characteristics of fruit from naranjilla segregants.

Group	Segregant	Weight (g)	Firmness (N)	Pulp Yield (%)	Peel + Seed (%)	Soluble Solids (°Brix)	Acidity (% Citric Acid)	Maturity Index	pH
1	T18	75.69 ± 0.31 b	50.98 ± 4.23 a	92.69 ± 0.0 b	7.31 ± 0.0 a	7.0 ± 0.0 c	2.19 ± 0.02 b	3.19 ± 0.04 b	3.24 ± 0.0 b
	TC2-67	78.04 ± 33.53 b	31.52 ± 4.09 a	93.89 ± 0.0 b	6.11 ± 0.0 a	7.8 ± 0.17 c	2.85 ± 0.02 ab	2.75 ± 0.06 b	3.03 ± 0.0 a
	T16	114.81 ± 0.42 a	17.49 ± 2.51 b	95.08 ± 0.0 a	4.92 ± 0.0 ab	8.5 ± 0.0 c	2.79 ± 0.0 ab	3.05 ± 0.0 b	3.22 ± 0.0 b
2	T12	76.55 ± 1.08 b	4.48 ± 0.64 c	92.72 ± 0.0 b	7.28 ± 0.0 a	10.0 ± 0.0 b	2.77 ± 0.01 ab	3.61 ± 0.01 b	3.3 ± 0.0 b
	T20	78.79 ± 0.91 b	4.27 ± 0.3 c	93.79 ± 0.0 b	6.21 ± 0.0 a	9.0 ± 0.0 b	2.01 ± 0.01 b	4.49 ± 0.02 ab	3.42 ± 0.0 c
	TP	90.97 ± 0.58 a	33.61 ± 2.95 a	95.98 ± 0.0 a	4.02 ± 0.0 ab	8.3 ± 0.33 c	2.77 ± 0.01 ab	3.01 ± 0.11 b	3.02 ± 0.0 a
3	T4	100.41 ± 0.41 a	33.31 ± 2.55 a	94.85 ± 0.0 ab	5.15 ± 0.0 ab	10.0 ± 0.0 b	2.75 ± 0.01 ab	3.64 ± 0.01 b	3.34 ± 0.0 b
	T22	70.97 ± 28.99 b	10.86 ± 3.5 b	95.78 ± 0.0 a	4.22 ± 0.0 ab	10.0 ± 0.0 b	2.66 ± 0.01 ab	3.77 ± 0.01 b	3.4 ± 0.0 c
	T24	57.51 ± 0.51 b	4.90 ± 0.1 c	92.65 ± 0.0 b	7.35 ± 0.0 a	11.5 ± 0.29 ab	1.74 ± 0.01 c	6.61 ± 0.15 a	3.43 ± 0.0 c
	P17	72.74 ± 1.37 b	4.64 ± 0.63 c	94.75 ± 0.0 ab	5.25 ± 0.0 ab	10.0 ± 0.0 b	2.92 ± 0.0 a	3.43 ± 0.01 b	3.36 ± 0.03 c
	P25	58.06 ± 0.53 b	2.25 ± 0.43 c	95.52 ± 0.0 a	4.48 ± 0.0 ab	12.3 ± 0.33 a	2.75 ± 0.01 ab	4.49 ± 0.13 ab	3.42 ± 0.04 c
	P30	64.47 ± 0.74 b	3.16 ± 0.51 c	97.27 ± 0.0 a	2.73 ± 0.0 b	9.7 ± 0.33 b	3.08 ± 0.01 a	3.14 ± 0.11 b	3.24 ± 0.06 b
4	P40	50.69 ± 0.31 b	3.48 ± 0.14 c	94.83 ± 0.0 ab	5.17 ± 0.0 ab	11.3 ± 0.33 ab	2.69 ± 0.01 ab	4.22 ± 0.14 ab	3.42 ± 0.04 c
5	*Sh*.3.89	41.48 ± 0.75 c	4.00 ± 0.17 c	95.75 ± 0.0 a	4.25 ± 0.0 ab	13.0 ± 0.0 a	2.38 ± 0.01 b	5.46 ± 0.02 a	3.54 ± 0.05 c
	28R3	51.96 ± 0.96 b	2.22 ± 0.21 c	96.99 ± 0.0 a	3.01 ± 0.0 b	11.3 ± 0.33 ab	3.37 ± 0.01 a	3.36 ± 0.11 b	3.29 ± 0.04 b
	P37	58.73 ± 0.73 b	1.78 ± 0.41 c	93.61 ± 0.0 b	6.39 ± 0.0 a	10.0 ± 0.0 b	2.74 ± 0.0 ab	3.64 ± 0.0 b	3.34 ± 0.05 b
	P28	57.85 ± 0.46 b	2.38 ± 0.48 c	95.87 ± 0.0 a	4.13 ± 0.0 ab	12.0 ± 0.0 a	2.84 ± 0.0 ab	4.23 ± 0.01 ab	3.39 ± 0.05 c
	P39	57.73 ± 0.37 b	2.71 ± 0.31 c	96.25 ± 0.0 a	3.75 ± 0.0 b	10.0 ± 0.0 b	2.94 ± 0.0 a	3.4 ± 0.0 b	3.39 ± 0.05 c
	P29	58.1 ± 0.1 b	2.48 ± 0.57 c	94.97 ± 0.0 ab	5.03 ± 0.0 ab	10.0 ± 0.0 b	2.89 ± 0.0 ab	3.46 ± 0.0 b	3.38 ± 0.04 c
	P18	53.85 ± 0.93 b	1.78 ± 0.36 c	96.82 ± 0.0 a	3.18 ± 0.0 b	10.0 ± 0.0 b	2.64 ± 0.01 ab	3.79 ± 0.01 b	3.31 ± 0.05 b

Means followed by different letters within columns differ significantly (Tukey, *p* ≤ 0.05).

**Table 2:** Mineral content of fruit from naranjilla segregants.

Group	Segregant	Ca (mg 100 g^−1^)	Mg (mg 100 g^−1^)	Zn (mg kg^−1^)	P (mg 100 g^−1^)	K (mg 100 g^−1^)	Fe (mg kg^−1^)	Na (mg 100 g^−1^)	Mn (mg kg^−1^)	Cu (mg kg^−1^)
1	T18	1.17 ± 0.01 a	0.17 ± 0.0 a	7.21 ± 0.14 bc	2.48 ± 0.0 b	2.92 ± 0.07 ab	30.17 ± 0.16 b	0.01 ± 0.0 c	0.0 ± 0.0 d	6.98 ± 0.20 a
	TC2-67	0.11 ± 0.0 b	0.18 ± 0.0 a	8.97 ± 0.37 b	2.51 ± 0.03 b	1.93 ± 0.01 c	24.09 ± 0.12 b	0.01 ± 0.0 c	4.12 ± 0.12 a	7.49 ± 0.23 a
	T16	0.06 ± 0.0 c	0.16 ± 0.0 a	23.49 ± 0.62 a	3.0 ± 0.02 a	2.02 ± 0.01 b	26.44 ± 0.54 b	0.02 ± 0.0 b	4.93 ± 0.1 a	8.00 ± 0.13 a
2	T12	0.09 ± 0.0 c	0.15 ± 0.0 b	21.11 ± 0.37 a	2.73 ± 0.04 b	2.80 ± 0.07 ab	25.32 ± 0.24 b	0.04 ± 0.0 a	0.01 ± 0.0 c	8.30 ± 0.25 a
	T20	0.58 ± 0.01 b	0.16 ± 0.0 b	7.16 ± 0.04 bc	2.14 ± 0.02 b	2.88 ± 0.05 ab	27.59 ± 0.7 b	0.03 ± 0.0 b	0.65 ± 0.01 c	12.00 ± 0.3 a
	TP	0.08 ± 0.0 c	0.14 ± 0.0 bc	7.76 ± 0.06 b	1.73 ± 0.04 c	1.90 ± 0.02 c	23.59 ± 0.13 b	0.01 ± 0.0 c	0.76 ± 0.02 c	8.03 ± 0.14 a
3	T4	0.07 ± 0.0 c	0.16 ± 0.0 a	10.06 ± 0.24 b	2.58 ± 0.0 b	2.57 ± 0.04 ab	21.74 ± 0.48 b	0.02 ± 0.0 b	1.51 ± 0.03 b	1.53 ± 0.03 c
	T22	0.06 ± 0.0 c	0.09 ± 0.0 d	8.83 ± 0.26 b	2.61 ± 0.04 b	3.41 ± 0.07 a	24.0 ± 0.74 b	0.02 ± 0.0 b	0.0 ± 0.0 d	4.48 ± 0.12 b
	T24	0.04 ± 0.0 d	0.13 ± 0.0 bc	4.52 ± 0.08 c	2.55 ± 0.0 b	2.77 ± 0.07 ab	32.84 ± 1.06 b	0.01 ± 0.0 c	0.0 ± 0.0 d	3.13 ± 0.06 c
	P17	0.03 ± 0.0 d	0.09 ± 0.0 d	5.98 ± 0.2 bc	2.69 ± 0.01 b	2.94 ± 0.06 a	19.23 ± 0.5 c	0.01 ± 0.0 c	0.76 ± 0.01 c	1.26 ± 0.00 c
	P30	0.08 ± 0.0 c	0.11 ± 0.0 c	7.88 ± 0.27 b	2.57 ± 0.0 b	2.85 ± 0.05 ab	19.31 ± 0.57 c	0.02 ± 0.0 b	3.39 ± 0.11 a	1.67 ± 0.04 c
	P25	0.06 ± 0.0 c	0.15 ± 0.0 b	10.13 ± 0.04 b	2.57 ± 0.03 b	2.05 ± 0.01 b	31.21 ± 0.37 b	0.02 ± 0.0 b	4.87 ± 0.05 a	0.0 ± 0.0 d
4	P40	0.06 ± 0.0 c	0.10 ± 0.0 c	8.7 ± 0.0 b	3.06 ± 0.02 a	2.93 ± 0.07 ab	45.43 ± 1.09 a	0.01 ± 0.0 c	3.90 ± 0.13 a	5.15 ± 0.12 b
5	*Sh*.3.89	0.07 ± 0.0 c	0.11 ± 0.0 c	9.34 ± 0.33 b	3.05 ± 0.06 a	2.14 ± 0.04 b	27.84 ± 0.5 b	0.01 ± 0.0 c	4.36 ± 0.1 a	7.93 ± 0.17 a
	28R3	0.04 ± 0.0 d	0.14 ± 0.0 bc	6.32 ± 0.15 bc	2.6 ± 0.05 b	2.31 ± 0.01 b	23.06 ± 0.37 b	0.01 ± 0.0 c	3.16 ± 0.11 a	3.08 ± 0.07 b
	P37	0.07 ± 0.0 c	0.15 ± 0.0 b	8.31 ± 0.1 b	2.86 ± 0.02 a	2.53 ± 0.02 ab	21.77 ± 0.53 c	0.01 ± 0.0 c	3.32 ± 0.12 a	6.70 ± 0.10 a
	P18	0.03 ± 0.0 d	0.14 ± 0.0 bc	7.13 ± 0.17 bc	2.96 ± 0.01 a	2.30 ± 0.06 b	17.98 ± 0.35 c	0.01 ± 0.0 c	4.28 ± 0.14 a	2.78 ± 0.08 c
	P28	0.03 ± 0.0 d	0.11 ± 0.0 c	6.72 ± 0.19 bc	2.54 ± 0.0 b	2.09 ± 0.04 b	19.02 ± 0.31 c	0.0 ± 0.0 c	3.04 ± 0.13 a	1.61 ± 0.02 c
	P39	0.04 ± 0.0 d	0.10 ± 0.0 c	6.59 ± 0.3 bc	2.61 ± 0.02 b	2.40 ± 0.0 b	22.04 ± 0.6 c	0.01 ± 0.0 c	0.47 ± 0.02 c	3.19 ± 0.09 b
	P29	0.04 ± 0.0 d	0.15 ± 0.0 b	8.49 ± 0.07 b	1.97 ± 0.03 c	2.12 ± 0.03 b	22.85 ± 0.71 c	0.0 ± 0.0 c	3.06 ± 0.08 a	1.88 ± 0.02 c

Means followed by different letters within columns differ significantly (Tukey, *p* ≤ 0.05).

**Table 3:** Antioxidant compounds and antioxidant capacity from naranjilla segregants.

Group	Segregant	Polyphenols (mg GAE g^−1^)	Flavonoids (mg catechin g^−1^)	Carotenoids (µg β-carotene g^−1^)	FRAP (µmol TE g^−1^)	ABTS (µmol TE g^−1^)	Vitamin C (mg 100 g^−1^)	Solanine (mg 100 g^−1^)	Chaconine (mg 100 g^−1^)
1	T18	6.65 ± 0.02 e	4.94 ± 0.06 e	94.30 ± 0.95 a	108.62 ± 0.89 c	106.60 ± 0.91 e	52.24 ± 0.22 bc	1.31 ± 0.00 d	3.65 ± 0.02 a
	TC2-67	9.71 ± 0.03 d	7.56 ± 0.02 d	49.78 ± 0.01 c	102.34 ± 0.30 c	154.36 ± 0.46 e	55.31 ± 0.67 b	1.65 ± 0.01 d	2.56 ± 0.01 a
	T16	9.86 ± 0.06 d	6.60 ± 0.04 d	73.16 ± 0.54 b	109.79 ± 0.39 c	152.92 ± 0.59 e	56.88 ± 0.76 b	1.91 ± 0.01 d	5.25 ± 0.00 b
2	T12	7.46 ± 0.06 e	4.01 ± 0.00 e	65.23 ± 0.14 b	107.15 ± 0.11 c	113.19 ± 0.69 e	40.35 ± 0.44 c	1.70 ± 0.03 d	5.27 ± 0.06 b
	T20	13.99 ± 0.02 c	8.94 ± 0.07 d	69.40 ± 0.61 b	166.80 ± 1.93 b	188.01 ± 0.91 d	49.31 ± 0.59 bc	1.21 ± 0.02 c	4.74 ± 0.01 b
	TP	9.26 ± 0.04 d	6.61 ± 0.02 d	61.98 ± 0.17 b	104.05 ± 0.68 c	147.98 ± 0.12 e	79.11 ± 0.22 a	1.13 ± 0.01 c	19.60 ± 0.01 c
3	T4	8.98 ± 0.08 d	7.34 ± 0.04 d	53.94 ± 0.37 c	131.83 ± 0.62 b	149.93 ± 1.22 e	19.39 ± 0.22 d	1.04 ± 0.01 c	3.54 ± 0.02 a
	T22	11.39 ± 0.16 c	7.73 ± 0.00 d	68.75 ± 0.51 b	158.94 ± 0.92 b	154.06 ± 0.60 e	54.43 ± 1.38 b	0.93 ± 0.03 c	4.03 ± 0.04 b
	T24	6.73 ± 0.10 e	3.50 ± 0.06 e	82.10 ± 0.20 a	107.82 ± 0.87 c	95.74 ± 0.89 e	50.67 ± 0.0 bc	1.17 ± 0.00 c	2.31 ± 0.02 a
	P25	9.28 ± 0.01 d	7.85 ± 0.04 d	55.20 ± 0.27 c	102.13 ± 1.31 c	212.75 ± 2.20 d	20.05 ± 0.44 d	0.15 ± 0.00 a	4.10 ± 0.01 b
	P30	16.71 ± 0.03 b	18.85 ± 0.07 b	62.34 ± 0.29 b	180.02 ± 0.66 b	378.50 ± 2.25 b	28.85 ± 0.44 cd	0.21 ± 0.00 a	5.26 ± 0.01 b
	P17	11.83 ± 0.05 c	11.35 ± 0.08 c	55.76 ± 0.63 c	144.16 ± 0.34 b	198.42 ± 0.81 d	18.28 ± 0.01 d	4.35 ± 0.01 f	5.27 ± 0.00 b
4	P40	28.03 ± 0.07 a	32.41 ± 0.13 a	66.83 ± 0.19 b	287.83 ± 0.65 a	552.77 ± 6.00 a	20.89 ± 0.44 d	0.47 ± 0.00 b	2.70 ± 0.00 a
5	*Sh*.3.89	8.56 ± 0.05 d	4.85 ± 0.04 e	50.98 ± 0.40 c	94.36 ± 0.08 c	131.57 ± 0.55 e	42.65 ± 0.38 c	1.64 ± 0.01 d	5.97 ± 0.00 b
	28R3	12.70 ± 0.04 c	9.71 ± 0.04 d	58.47 ± 0.18 b	178.16 ± 2.32 b	201.68 ± 0.93 d	30.47 ± 0.0 cd	1.33 ± 0.01 c	4.63 ± 0.00 b
	P37	16.26 ± 0.03 b	14.07 ± 0.07 c	62.98 ± 0.66 b	230.70 ± 0.84 a	256.28 ± 0.28 c	41.72 ± 0.38 c	2.06 ± 0.00 e	2.93 ± 0.00 a
	P18	12.17 ± 0.05 c	11.69 ± 0.15 c	64.93 ± 0.75 b	147.57 ± 0.45 b	207.42 ± 1.08 d	41.71 ± 0.44 c	0.73 ± 0.00 b	4.07 ± 0.00 b
	P28	9.51 ± 0.02 d	8.50 ± 0.00 d	46.40 ± 0.19 c	117.77 ± 1.04 c	160.26 ± 2.83 e	50.11 ± 0.59 bc	1.52 ± 0.00 d	2.30 ± 0.00 a
	P39	13.15 ± 0.09 c	12.93 ± 0.02 c	50.28 ± 0.81 c	140.33 ± 0.12 b	269.34 ± 1.05 c	22.1 ± 0.44 d	0.41 ± 0.00 b	4.07 ± 0.01 b
	P29	8.92 ± 0.14 d	6.45 ± 0.08 d	48.38 ± 0.75 c	86.75 ± 0.36 c	161.31 ± 2.95 e	25.67 ± 0.0 cd	0.16 ± 0.00 a	2.21 ± 0.00 a

Means followed by different letters within columns differ significantly (Tukey, *p* ≤ 0.05). FRAP: Ferric Reducing Antioxidant Power; ABTS: 2,2′-azinobis(3-ethylbenzothiazoline-6-sulfonic acid); GAE: gallic acid equivalent.

**Table 4:** Z-score values for the fruit quality traits in a *S. quitoense* breeding population.

Segregant	ABTS	SS (°Brix)	Pulp Yield (%)	Z ABTS	Z SS	Z Pulp Yield	Composite Index
P40	552.77	12	95.08	3.407	1.258	0.114	4.779
P28	165.16	12	95.78	−0.333	1.258	0.619	1.544
P25	212.75	13	94.75	0.126	1.917	−0.510	1.534
P39	269.34	10	95.98	0.672	−0.060	0.762	1.374
T24	95.74	12	96.25	−1.003	1.258	0.961	1.217

ABTS: 2,2′-azinobis(3-ethylbenzothiazoline-6-sulfonic acid); SS: soluble solids; Z: Z-score.

**Table 5:** Improved naranjilla (*S. quitoense*) lines.

No.	Segregant	Genealogy (Filial)
1	T4	*S. quitoense* var. *quitoense* cv. Baeza
2	T12	*S. ves/S. qui//S. qui* cv. Bae*///S. hir/S. qui* cv. Tan *(selec 12-1)*
3	T18	*S. ves/S. qui//S. qui* cv. Bae*///S. hir/S. qui* cv. Tan *(selec 18-2)*
4	T20	*S. quitoense* cv. Tandapi*//S. hirtum/S. quitoense* cv. Tandapi *(selec 2-20-1)*
5	T22	*S. vestissimum/S. quitoense//S. quitoense* cv. Baeza*///S. hirtum/S. quitoense* cv. Tandapi
6	T24	*S. quitoense* cv. Tandapi*//S. hir/S. quitoense* cv. Tandapi *(selec 38-102-1)*
7	TC2-67	*S. hyporhodium/S. qui* cv. Baeza *(selec 2-67-1)*
8	T16	*S. vestissimum/S. quitoense/S. quitoense* cv. Baeza *(selec 16-16-1)*
9	TP	*S. quitoense/S. sessiliflorum*
10	*Sh*.3.89	*Solanum hirtum (selec 3-89)*
11	P28	*S. quitoense G2p39 x S. hiporhodium*
12	P37	*S. quitoense G2p46 x S. hiporhodium*
13	P28R3	*S. quitoense G2p37 x S. hiporhodium*
14	P17	*S. quitoense G2p45 x S. hiporhodium*
15	P18	*S. quitoense G2p45 x S. hiporhodium*
16	P39	*S. quitoense G2p46 x S. hiporhodium*
17	P40	*S. quitoense G2p46 x S. hiporhodium*
18	P25	*S. quitoense G2p39 x S. hiporhodium*
19	P30	*S. quitoense G8p31x S. hiporhodium*
20	P29	*S. quitoense G2p46 x S. hiporhodium*

## Data Availability

The original contributions presented in this study are included in the article/Supplementary Materials. Further inquiries can be directed to the corresponding author.

## Supplementary Materials

The following supporting information can be downloaded at: https://www.mdpi.com/article/10.3390/molecules31132217/s1, Table S1: Z-score ranking of the evaluated naranjilla segregants.
