# Supplementary material for: Phytochemical Characterisation of Naranjilla (Solanum quitoense Lam.) Segregants from Interspecific Crosses Within Section Lasiocarpa
Source: Molecules. 2026 Jun 24;31(13):2217. doi: 10.3390/molecules31132217 (PMC13362583; doi:10.3390/molecules31132217)
Supplement: Supplementary file 1 [file molecules-31-02217-s001.zip › molecules-4346399-supplementary.pdf]

Table S1. Z-score ranking of the evaluated naranjilla segregants.

Segregants	ABTS	SS (°Brix)	pulp_yield	Z_ABTS	Z_brix	Z_yield	Composite_index
P40	552.77	12	95.0783	3.407	1.258	0.114	4.779
P40	563.17	11	95.0783	3.507	0.599	0.114	4.220
P40	542.38	11	95.0783	3.306	0.599	0.114	4.020
P17	165.16	12	95.7767	-0.333	1.258	0.619	1.544
P29	212.75	13	94.2143	0.126	1.917	-0.510	1.534
P17	160.26	12	95.7767	-0.380	1.258	0.619	1.497
P17	155.37	12	95.7767	-0.427	1.258	0.619	1.450
P25	271.16	10	95.9752	0.690	-0.060	0.762	1.392
P25	269.34	10	95.9752	0.672	-0.060	0.762	1.374
P25	267.53	10	95.9752	0.655	-0.060	0.762	1.357
T24	95.74	12	96.2500	-1.003	1.258	0.961	1.217
P29	216.56	12	94.2143	0.163	1.258	-0.510	0.911
T24	94.20	11.5	96.2500	-1.017	0.928	0.961	0.872
P29	208.95	12	94.2143	0.090	1.258	-0.510	0.838
P28	382.41	10	93.6100	1.763	-0.060	-0.947	0.756
P28	378.50	10	93.6100	1.725	-0.060	-0.947	0.718
T12	114.39	10	96.9900	-0.823	-0.060	1.496	0.613
T12	113.19	10	96.9900	-0.834	-0.060	1.496	0.601
T12	112.00	10	96.9900	-0.846	-0.060	1.496	0.590
T24	97.28	11	96.2500	-0.988	0.599	0.961	0.572
T20	189.59	9	96.8200	-0.097	-0.720	1.373	0.556
T20	188.01	9	96.8200	-0.112	-0.720	1.373	0.541
T20	186.43	9	96.8200	-0.128	-0.720	1.373	0.526
TP	147.98	9	97.2700	-0.499	-0.720	1.698	0.480
P30	166.42	10	95.7527	-0.321	-0.060	0.602	0.221
P30	161.31	10	95.7527	-0.370	-0.060	0.602	0.171
P30	156.20	10	95.7527	-0.419	-0.060	0.602	0.122
P28	374.60	9	93.6100	1.688	-0.720	-0.947	0.021
TP	148.18	8	97.2700	-0.497	-1.379	1.698	-0.178
TP	147.77	8	97.2700	-0.501	-1.379	1.698	-0.181
Sh.3.89	132.52	13	92.7238	-0.648	1.917	-1.587	-0.317
P28R3	256.76	10	93.7909	0.551	-0.060	-0.816	-0.325
Sh.3.89	131.57	13	92.7238	-0.657	1.917	-1.587	-0.326
P28R3	256.28	10	93.7909	0.546	-0.060	-0.816	-0.330
P37	201.68	12	92.6905	0.020	1.258	-1.611	-0.333

P28R3	255.79	10	93.7909	0.542	-0.060	-0.816	-0.335
Sh.3.89	130.62	13	92.7238	-0.666	1.917	-1.587	-0.336
T22	155.10	10	94.8300	-0.430	-0.060	-0.065	-0.555
T22	154.06	10	94.8300	-0.440	-0.060	-0.065	-0.565
T4	152.04	10	94.8500	-0.459	-0.060	-0.051	-0.570
T22	153.02	10	94.8300	-0.450	-0.060	-0.065	-0.575
T4	149.93	10	94.8500	-0.480	-0.060	-0.051	-0.591
T4	147.81	10	94.8500	-0.500	-0.060	-0.051	-0.611
P39	209.30	10	93.8915	0.093	-0.060	-0.743	-0.711
P39	207.42	10	93.8915	0.075	-0.060	-0.743	-0.729
P39	205.54	10	93.8915	0.057	-0.060	-0.743	-0.747
P37	203.28	11	92.6905	0.035	0.599	-1.611	-0.977
P37	200.08	11	92.6905	0.004	0.599	-1.611	-1.008
T16	153.93	8.5	95.5200	-0.441	-1.049	0.434	-1.057
T16	152.92	8.5	95.5200	-0.451	-1.049	0.434	-1.067
T16	151.90	8.5	95.5200	-0.461	-1.049	0.434	-1.077
P18	199.83	10	92.6470	0.002	-0.060	-1.643	-1.701
P18	198.42	10	92.6470	-0.012	-0.060	-1.643	-1.715
P18	197.01	10	92.6470	-0.025	-0.060	-1.643	-1.728
TC2-67	154.36	8	94.9700	-0.437	-1.379	0.036	-1.780
TC2-67	153.57	8	94.9700	-0.445	-1.379	0.036	-1.788
TC2-67	155.16	7.5	94.9700	-0.429	-1.709	0.036	-2.102
T18	108.17	7	94.7500	-0.883	-2.038	-0.123	-3.044
T18	106.60	7	94.7500	-0.898	-2.038	-0.123	-3.059
T18	105.03	7	94.7500	-0.913	-2.038	-0.123	-3.074
